# Gastrointestinal signals in supplemented media reveal a role in adherence for the *Shigella flexneri sap* autotransporter gene

**DOI:** 10.1080/19490976.2024.2331985

**Published:** 2024-03-28

**Authors:** Yrvin León, Raphael Honigsberg, David A. Rasko, Christina S. Faherty

**Affiliations:** a Mucosal Immunology and Biology Research Center, Division of Pediatric Gastroenterology and Nutrition, Massachusetts General Hospital, USA; b Department of Pediatrics, Harvard Medical School, Boston, MA, USA; c École Normale Supérieure Paris-Saclay, Département d’Enseignement et de, Recherche de Biologie, Université Paris-Saclay, Gif-sur-Yvette, France; d Institute for Genome Sciences, Center for Pathogen Research, Department of Microbiology and Immunology, University of Maryland School of Medicine, Baltimore, MD, USA

**Keywords:** *Shigella flexneri*, adherence factors, bile salts, glucose, autotransporter, *sap*

## Abstract

*Shigella flexneri* causes severe diarrheal disease worldwide. While many aspects of pathogenesis have been elucidated, significant knowledge gaps remain regarding the role of putative chromosomally-encoded virulence genes. The uncharacterized *sap* gene encoded on the chromosome has significant nucleotide sequence identity to the fluffy (*flu*) antigen 43 autotransporter gene in pathogenic *Escherichia coli*. Here, we constructed a Δ*sap* mutant in *S. flexneri* strain 2457T and examined the effects of this mutation on bacterial cell aggregation, biofilm formation, and adherence to colonic epithelial cells. Analyses included the use of growth media supplemented with glucose and bile salts to replicate small intestinal signals encountered by *S. flexneri*. Deletion of the *sap* gene in 2457T affected epithelial cell adherence, resulted in quicker bacterial cell aggregation, but did not affect biofilm formation. This work highlights a functional role for the *sap* gene in *S. flexneri* pathogenesis and further demonstrates the importance of using relevant and appropriate gastrointestinal signals to characterize virulence genes of enteropathogenic bacteria.

## Introduction

*Shigella* is a genus of Gram-negative, facultative anaerobic bacteria that includes four species and more than 50 serotypes. *Shigella* is an important diarrheal pathogen afflicting the globe, particularly in lower- and middle-income countries.^1^ The infectious dose is very low, in which as little as 10 to 100 bacteria can cause infection.^2^ The Global Burden of Disease estimates that *Shigella* is the second most common cause of diarrheal deaths, with approximately 165,000 annual deaths worldwide, particularly in children, the elderly, and immunocompromised patients.^3,4^ This disease burden is further complicated by an increasing prevalence of antibiotic resistance and the lack of an effective, approved vaccine.^5^

The primary mode of transmission of *Shigella* is through the fecal-oral route typically by ingestion of contaminated food or water.^1^ After *Shigella* survives the acidic conditions in the stomach, the bacteria resist bile salts during transit of the small intestine.^6,7^ Bile salts are the principal components of bile, which aids in digestion by absorbing lipids and fat-soluble vitamins. Moreover, bile salts have antimicrobial properties that compromise the membrane of bacteria.^8^ However, *Shigella* and many other enteric pathogens resist bile salts and alter transcriptional profiles in response to exposure.^6,7^ After reaching the colonic epithelium, *Shigella* induces transcytosis of M cells, specialized antigen-presenting cells of the colonic epithelium, to invade epithelial cells at the basolateral pole.^1^ Invasion, cytoskeleton modifications, and host cell signaling manipulation require a series of protein effectors that are delivered into the cytoplasm of the host cell by the needle-like type-III secretion system (T3SS).^9^ As infection proceeds, interleukin-8 (IL-8) is secreted from infected epithelial cells, resulting in a significant recruitment of neutrophils to the site of infection, ultimately leading to tissue destruction that exacerbates the symptoms of the disease.^1,9^ Despite extensive research on virulence genes encoded on the *Shigella* virulence plasmid, the role of several putative virulence genes encoded on the chromosome remains unknown. Characterization of these additional chromosomally-encoded virulence genes may help advance our understanding of infection and illuminate new therapeutic strategies to eradicate shigellosis.

Most *S. flexneri* serotype 2a strains harbor the *she* pathogenicity island (PAI), which is a genomic region of 31 open reading frames encoded on the chromosome.^10^ There is an unusual clustering of three genes that each encode proteins classified as autotransporters, including the putative autotransporter *sap* (*S**higella* autotransporter-like protein) gene.^10,11^ Autotransporters or proteins of the type-V secretion system, are large proteins consisting of an N-terminal signal sequence and a passenger domain that is secreted to the bacterial outer surface through a unique secretion mechanism utilizing the C-terminal β-barrel domain of the protein that embeds into the outer membrane of the bacterial cell.^12^ The passenger domain is the structural motif that harbors the biological function and is often directly involved in virulence mechanisms such as bacterial cell aggregation, biofilm formation, adhesion to epithelial cells, host immune evasion, and other cytopathic effects.^13–17^ The *sap* gene has 99% nucleotide identity to the *Escherichia coli flu* gene that encodes the autotransporter antigen 43 (Ag43).^10,14^ Ag43 in *E. coli* has been characterized to have several functions associated with virulence in different *E. coli* pathovars, including bacterial cell aggregation and alterations of bacterial shape in enterohemorrhagic *Escherichia coli* (EHEC) serotype O157:H7.^18^ Ag43 also contributes to the adhesion of uropathogenic *Escherichia coli* (UPEC) strain CFT073, which increases long-term persistence of the pathogen in the bladder of a mouse model of urinary tract infection.^19^ Finally, Ag43 has been shown to enhance the survival of *E. coli* by forming bacterial aggregates inside neutrophils.^20^

Despite the unknown function of the *sap* gene in *S. flexneri* 2457T serotype 2a, *in silico* analysis identified a highly immunogenic peptide within the Sap protein, which was recently included in a multi-epitope recombinant fusion vaccine candidate with two additional immunogenic peptides, the autotransporters Pic and SigA from *S. flexneri* 2457T. Inclusion of the Sap peptide was due to the autotransporter-like properties and the predicted outer membrane localization of the protein.^11^ Vaccine efficacy was subsequently examined in an intranasal murine infection model and found to increase construct-specific antibody (serum IgG and fecal IgA) and cytokine (TNF-α, IL-17, and IFN-γ) titers. The vaccine candidate conferred 100% protection against *S. flexneri* 2457T in the murine model, highlighting the importance of mucosal immunity activation, of which the autotransporters appear to be a critical component.^21^ Since the function of the *sap* gene in *S. flexneri* remains largely unknown, the goal of this study was to evaluate a *sap* mutant to determine a possible role in *S. flexneri* virulence.

## Results

### *Inspection of the* sap *coding region with previous RNA-sequencing data and Δ*sap *mutant construction*

**Figure 1.**
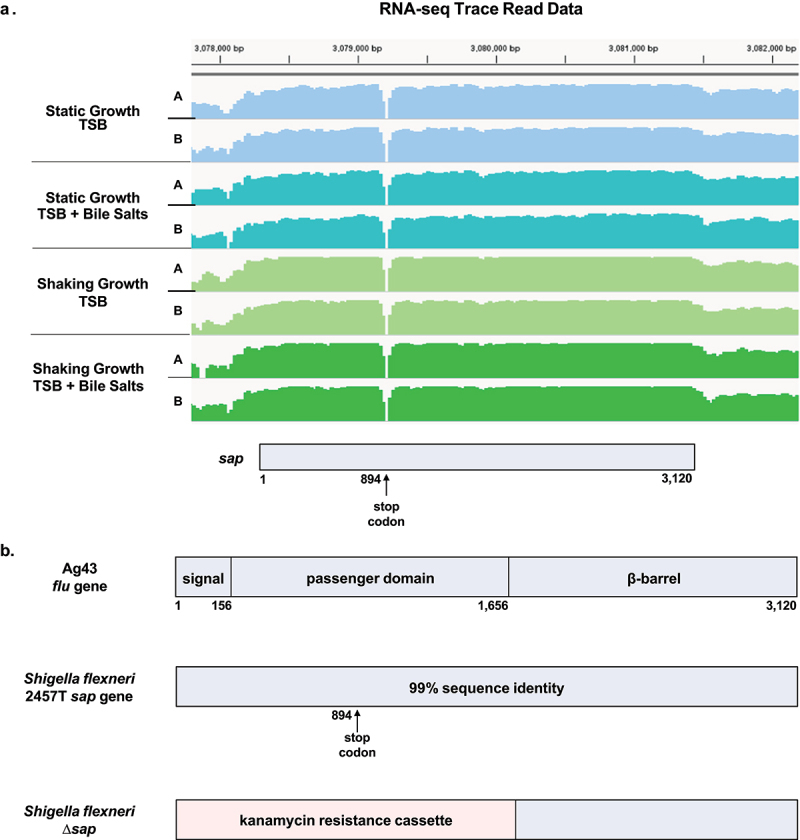
Analyses of the *sap* coding region and gene deletion strategy. (**a**) the *sap* coding region, which is located at the twentieth open reading frame (orf) within the *she* pathogenicity island, was analyzed using previous RNA-sequencing gene expression data.^6^ RNA was extracted from *S. flexneri* 2457T cultures grown in TSB with and without bile salts under both shaking and static growth conditions, each with technical duplicate cultures (A and B). The RNA-seq trace read data were generated using the integrative viewer (IGV) software (version 2.14). There were no significant expression changes in the *sap* gene in the different growth conditions. The corresponding *sap* coding region is provided at the bottom of the diagram, and the gap in sequencing corresponds to the annotated stop codon at position 894. (**b**) Comparison of the *sap* gene sequence with the Ag43 *flu* gene sequence of *E. coli* ^14^ indicated 99% identity of the signal sequence (nucleotides 1–156), the passenger domain (nucleotides 157–1,656) and the β-barrel domain (nucleotides 1,657–3,120). The only sequence difference is the stop codon at position 894 in the *S. flexneri* 2457T sequence. The lambda red linear recombination method was used to replace the 5’ region of the *sap* gene, specifically the signal sequence and the passenger domains (1.7 kb), with a kanamycin resistance cassette. The mutation was confirmed by PCR analysis of the wild type and ∆*sap* kanamycin resistant colonies.

Since the *sap* gene (S3195) in *S. flexneri* serotype 2a strain 2457T (GenBank Accession Number AE014073.1) is currently annotated as a pseudogene, we inspected the *sap* coding region using previously generated data from high-throughput RNA-sequencing-based gene expression analyses.^6^ The gene expression data were obtained from *S. flexneri* 2457T grown in tryptic soy broth (TSB) with and without bile salts under both shaking and static growth conditions at 37°C.^6^ The analyses of *sap* gene expression were consistent in the four different growth conditions, and there were no significant differences among the gene expression patterns (Figure 1(a)). Sequence comparison of the *sap* gene with the *E. coli* Ag43 *flu* gene indicated 99% nucleotide sequence identity.^14^ However, in *S. flexneri* 2457T, there is a stop codon in the passenger domain of the *sap* gene at nucleotide position 894 that also corresponds with a break in the RNA-seq transcript reads (Figure 1(a)). We further analyzed the sequence region and discovered a putative Rho-dependent termination sequence with the RhoTermPredict algorithm.^22^ A putative Rho utilization (RUT) site at nucleotides 874–952 and a corresponding pause site with appropriate hairpin structures in the 150 nucleotides immediately downstream of the RUT site were detected,^22,23^ which could explain the transcriptional profile and break in the RNA-seq reads.

We utilized the stop codon to evaluate sequence divergence of the *sap* gene across different *S. flexneri* 2a isolates. Interestingly, the *S. flexneri* serotype 2a strain YSH6000T *sap* gene homolog (*orf20*) does not contain a stop codon (GenBank Accession Number AF200692.2); however, the *sap* gene (SF2990) in *S. flexneri* serotype 2a strain 301 (GenBank Accession Number AE005674.2) is annotated as an intact gene from nucleotide 1 to the same premature stop codon, while the C-terminal domain is annotated as a separate outer membrane fluffing protein gene (SF2991). Based on the variable annotations across the *S. flexneri* serotype 2a strains, we evaluated the *sap* region in the global collection of *S. flexneri* isolates analyzed in the GEMS collection (Table 1).^24^ In this global collection of *S. flexneri* isolates, only 150 (19%) of the 806 sequenced strains encode the *sap* gene, most of which (17%, 137/806) contain the premature stop codon. An additional 36% of the isolates (291/806) contained a sequence that was significantly similar to the *sap* gene region but divergent (35–70% nucleotide identity), whereas 45% (365/806) showed little or no primary sequence similarity to the *sap* gene (<30% nucleotide identity). This *in silico* analysis highlights the significant *sap* gene sequence divergence across the *S. flexneri* species.

**Table 1.** The presence of the *sap* gene in the global collection of *S. flexneri* isolates (GEMS).

Description of Isolates	Number of Isolates (% of total)
Full length gene present	150 (19%)
With the stop codon	137 (17%)
Without the stop codon	13 (2%)
Limited sequence similarity	291 (36%)
No significant *sap* sequence homology	365 (45%)
**Total isolates**	**806**

To further evaluate the potential functions of the *sap* gene in the pathogenesis of *S. flexneri* strain 2457T, we used the lambda red linear recombination method^25^ to disrupt the 5’ end of the *sap* gene, which encodes the N-terminal signal sequence and the passenger domain of the putative Sap protein. This 1.7 kb region was replaced with a kanamycin antibiotic resistance cassette (Figure 1(b)), which was verified by PCR. The ∆*sap* mutant was also sequenced to ensure the insertion of the kanamycin cassette was the only significant genetic change to the strain. Subsequent growth curve analyses of the Δ*sap* mutant compared to wild type 2457T in TSB media with and without 0.4% weight/volume (w/v) bile salts indicated no effect of the mutation on the growth of the bacteria or bile salts resistance.

### *The effect of the deletion of the* sap *passenger domain on bacterial cell aggregation and biofilm formation in* S. flexneri

Bacterial cell aggregation, or autoaggregation, is a phenotype in which bacteria of the same clone or species form multicellular aggregates. This process is typically mediated by self-recognizing proteins that enable bacterial cells to bind together, or through exopolysaccharides such as lipopolysaccharides (LPS) in Gram-negative bacteria.^26^ Ag43 in *E. coli* is the most common autotransporter protein involved in this self-recognizing mechanism.^14,27^ Ag43 is also associated with biofilm formation in some strains of *E. coli*.^14,18^ Biofilms are organized bacterial communities that begin with bacterial attachment and microcolony formation followed by the expression of an extracellular polymeric substance (EPS) matrix, which allows bacteria to selectively interact with the extracellular environment and resist harsh conditions.^28^ Previous literature has not evaluated the ability of *S. flexneri* to use self-recognizing proteins for autoaggregation. However, cellular aggregation and biofilm formation occur after bile salts and glucose exposure during subculture. The structural proteins of the long polar fimbriae (LpfA), type 1 fimbriae (FimA), and curli (CsgAB) adherence factors, which are also expressed in the presence of both glucose and bile salts, facilitate the adherence stage of biofilm formation in *S. flexneri*.^29^ Biofilm formation is further enhanced by robust EPS matrix production starting at approximately 4 h of subculture.^6,30^ Furthermore, recent work has demonstrated that the *S. flexneri* autotransporter protein IcsA, a protein that mediates actin-based motility in the cytoplasm of infected host cells,^9^ facilitates aggregation and bacterial cell-to-cell contact during biofilm formation.^31^ Importantly, these phenotypes do not occur in Luria broth (LB) media, which is the most common enteric growth media.^6^ Therefore, and given the function of Ag43 in *E. coli*, we wanted to evaluate the ∆*sap* mutant in autoaggregation and biofilm formation assays.

**Figure 2.**
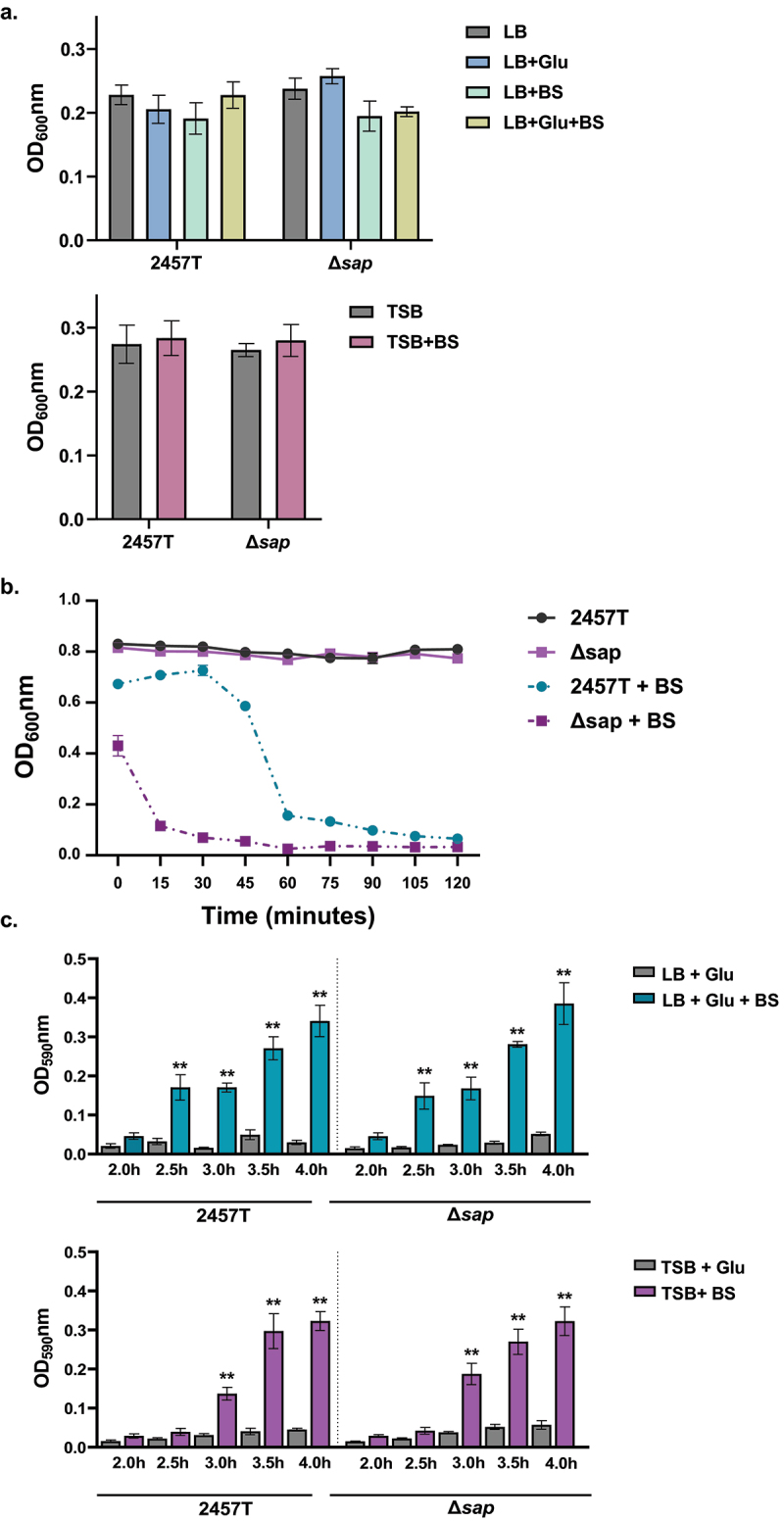
Bacterial cell aggregation and biofilm formation analyses. Overnight cultures of *S. flexneri* wild type strain 2457T and the Δ*sap* mutant were standardized and inoculated into different media formulations at 1:50 dilutions, either LB supplemented with glucose (glu) and/or bile salts (BS) or TSB supplemented with bile salts, and subcultured. All data are plotted as the average of three biological independent experiments, ± the standard error of the mean. (**a**) To measure bacterial cell aggregation prior to EPS matrix formation, subcultures were grown in shaking conditions until growth reached an OD_600_ of 0.7. Bacterial cultures were then centrifuged, resuspended in 2 ml of 1X PBS, and placed on the benchtop for 18 h. The OD_600_ was measured 0.5 cm from the top of the supernatants at the beginning (time 0) and after the 18 h incubation. Bacterial cell aggregation was measured as the final OD_600_ readings at 18 h subtracted from the initial OD_600_ readings for each condition. No significant differences were detected in any of the conditions tested. (**b**) To measure bacterial cell aggregation after EPS matrix formation, subcultures were grown as noted above for 4 hours in TSB with and without bile salts supplementation. While there was no autoaggregation in 2457T or the Δ*sap* mutant in TSB, both strains aggregated in TSB + BS (*p* < 0.01 for all time points), and the Δ*sap* mutant aggregated quicker compared to 2457T (*p* ≤0.001 for all time points). Analyses were performed in LB + glu ± BS and the same results were obtained, but the higher glucose concentrations resulted in the Δ*sap* mutant aggregating within five minutes (data not shown). (**c**) Biofilm formation was monitored every 30 minutes between hours 2 to 4 of incubation to capture the early phase of formation. There were significant increases in the biofilm formation in all media containing both glucose and bile salts in 2457T and the Δ*sap* mutant (*p* < 0.005) as indicated by the asterisks. EPS matrix production started at 2.5 hours for LB + glu + BS and at 3.0 hours for TSB + BS in which the delay is due to the lower glucose concentration. There were no significant differences between 2457T and the Δ*sap* mutant.

Starting with the self-recognizing mechanism of autoaggregation, this phenotype was examined using a clumping assay that measures bacterial aggregates at the bottom of culture tubes.^32^ To properly consider autoaggregation both before and after biofilm formation, wild type 2457T and the Δ*sap* mutant were cultured in LB media supplemented with or without glucose and/or bile salts, as well as TSB supplemented with or without bile salts at different time points. Given the range of glucose concentrations in the small intestine,^33,34^ LB media supplemented with 2% w/v glucose was used to replicate high glucose concentrations, while TSB media already contains added glucose at 0.5% w/v and replicates low glucose concentrations as previously described.^6^ Under glucose and bile salts subculturing conditions of approximately 2.5 to 3 h (depending on the glucose concentration), EPS matrix production of the biofilm is not yet detected.^6^ Thus, autoaggregation prior to EPS matrix production was examined in all media types. At this early time point, autoaggregation was not observed for either 2457T or the ∆*sap* mutant, and there were no significant differences between the strains (Figure 2(a)). However, after EPS matrix production started, autoaggregation was observed. Interestingly, the ∆*sap* mutant aggregated quicker relative to 2457T (Figure 2(b), note the different decreases in OD_600_ readings), indicating that autoaggregation during biofilm formation may be independent of or inhibited by the *sap* gene product. For complete biofilm formation, we compared the abilities of wild type 2457T and the ∆*sap* mutant to form biofilms during incubation times of 2–4 h using our 96-well plate assay as previously described.^6,30^ These results demonstrated that biofilm formation progressively increased after bile salts exposure in the presence of glucose, for both 2457T and the ∆*sap* mutant. There were no significant differences between the strains during the time periods examined (Figure 2(c)), or longer incubation times up to 24 h (data not shown). These data indicate that the *sap* gene product is not directly involved in autoaggregation or biofilm formation, including the early stages of formation, in *S. flexneri* strain 2457T.

### *The* sap *gene product has a role in* S. flexneri *adherence to HT-29 intestinal epithelial cells*

**Figure 3.**
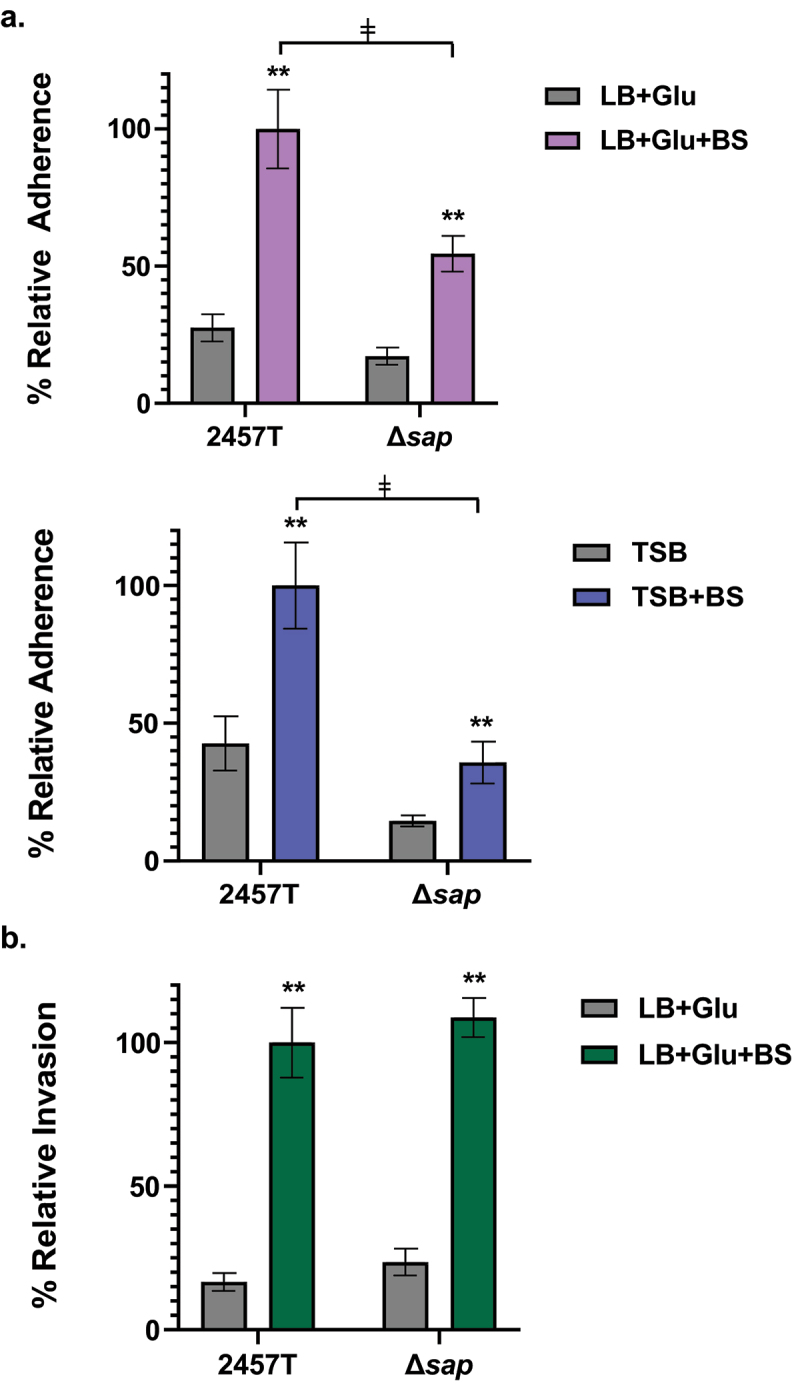
Analysis of the ∆*sap* mutant in adherence to and invasion of HT-29 epithelial cells. Infection analyses were performed following bacterial subculture in LB media supplemented with glucose (glu), with or without bile salts (BS), as well as TSB media with or without bile salts. The average percent recoveries ± standard error of the mean from three biological replicates are plotted relative to the recovery rate for 2457T from the bile salts subcultures (set at 100%). (**a**) In adherence assays following subculture in either LB + glu ± BS or TSB ± BS, the adherence rates were induced for both wild type 2457T and the Δ*sap* mutant following bile salts exposure (**, *p <* 0.005). However, the Δ*sap* mutant had lower adherence following subculture in bile salts compared to 2457T in the same condition (ǂ, *p* < 0.005). There were no significant differences in adherence between 2457T or the Δ*sap* mutant following subculturing in media without bile salts. While there is a decrease in adherence in the Δ*sap* mutant relative to 2457T following TSB subculture without bile salts, the difference was not statistically significant. (**b**) For invasion, there were no significant differences between 2457T and the Δ*sap* mutant following subculture in LB + glu ± BS. Both strains had significant increases in invasion following exposure to bile salts (**, *p* < 0.005).

Colonization of the gastrointestinal tract by commensal or pathogenic bacteria is typically mediated by surface structures such as pili or fimbriae to initiate contact with host cells.^35^ Ag43 has been shown to facilitate adherence of *E. coli*.^19^ *S. flexneri* adherence to colonic epithelial cells is mediated by surface proteins, including LpfA, FimA, and CsgAB, as well as the T3SS effectors OspE1 and OspE2, in which adherence and surface localization of these proteins are induced after exposure to physiological concentrations of bile salts and glucose.^6,29,36^ To determine a possible role of the *sap* gene product in the adherence of *S. flexneri* to colonic epithelial cells, adherence assays with HT-29 cells were performed for wild type 2457T and the Δ*sap* mutant. The adherence of both bacterial strains was evaluated following bacterial subculturing in high (LB + glucose) and low (TSB) glucose concentrations, both with or without bile salts. It is essential to note that glucose and bile salts are important small intestinal signals. For subsequent infection analyses of colonic epithelial cells, the bacteria were washed and resuspended in standard tissue culture media to mimic the transition into the colon prior to applying the bacteria to the HT-29 cells.^6,29,36^ Our results demonstrate that wild type 2457T and the Δ*sap* mutant both displayed increased adherence after bile salts exposure in both media types relative to media without bile salts (*p* < 0.005; Figure 3(a,b)). Interestingly, despite the same pattern of induced adherence following bile salts exposure, the adherence rate of the Δ*sap* mutant relative to wild type 2457T was significantly lower, by approximately 50%, only when the bacteria were subcultured in bile salts (*p* < 0.005; Figure 3(a,b)). These data indicate that the *sap* gene product has a role in the adherence phenotype of *S. flexneri* to HT-29 cells, which was detected only after subculturing the bacteria in the presence of small intestinal signals.

The invasion of colonic epithelial cells is a critical step in the *S. flexneri* lifecycle, in which entry into the cellular cytoplasm is required to establish a replicative niche of *S. flexneri*. Given the importance of host cell invasion, we evaluated the ability of the Δ*sap* mutant to invade the epithelial cells. For Ag43, there is no evidence that this autotransporter plays a role in invasion,^37^ especially since most *E. coli* pathovar isolates are noninvasive.^38^ Thus, we hypothesized that the mutation would not affect invasion of *S. flexneri*. To test this hypothesis, we performed an invasion assay with HT-29 cells following the same subculture conditions as the adherence assays.^6,39^ The bacteria were centrifuged onto the HT-29 cells to facilitate bacterial contact with the epithelial cells and bypass the adherence step.^39^ As expected, bile salts exposure increased the invasion rates^40,41^ for both wild type 2457T and the ∆*sap* mutant, but there were no statistical differences (Figure 3(c)), indicating that the *sap* gene is not directly involved in the invasion of *S. flexneri* into colonic epithelial cells.

## Discussion

Bacillary dysentery caused by *S. flexneri* is a bloody diarrheal disease that is the second leading cause of mortality.^3^ Despite understanding important concepts in pathogenesis, particularly regarding host cell invasion and immune evasion, many gaps in knowledge remain. The chromosomal *she* PAI of *S. flexneri* harbors autotransporter genes such as *sap* that encode highly immunogenic proteins, particularly the amino acid sequence of the passenger domain.^11^ Given the immunogenic effect of a recombinant protein that included a Sap peptide in a *S. flexneri* vaccine candidate,^21^ we sought to study the role of the *sap* gene in *S. flexneri* pathogenesis. We evaluated the ∆*sap* mutant in autoaggregation, biofilm formation, and adherence assays due to the homology of *sap* to the *flu* gene encoding the autotransporter Ag43.^10,14–16,19^ Furthermore, since many of these phenotypes require supplemented laboratory media for the appropriate analysis of *S. flexneri*,^6,29^ we performed our analyses with standard laboratory media, as well as media supplemented with bile salts and glucose based on our previous work.

Autoaggregation is defined as multicellular clumps of bacteria that confer protection against environmental stresses or host responses, and can also help initiate biofilm formation in a self-recognizing manner.^26^ Thus, it is important to evaluate both autoaggregation and biofilm formation in the ∆*sap* mutant. Our analyses demonstrated that the *S. flexneri sap* gene product is not directly involved in either process. We tested for autoaggregation at 2 h of subculturing, which is prior to when the EPS matrix is secreted during biofilm formation (see the increases in OD_590_ readings in Figure 2(c)) at 2.5 and 3 h, depending on the glucose concentration and our previous work.^6,29^ In experiments in which autoaggregation was tested after secretion of the EPS matrix, the ∆*sap* mutant aggregated quicker than wild type 2457T (Figure 2(b)). Given that *sap* gene expression was not affected by bile salts exposure (Figure 1(a)), we were not surprised by the lack of a direct effect of the *sap* deletion on autoaggregation or biofilm formation, but we were surprised by the quicker aggregative phenotype of the Δ*sap* mutant following EPS secretion. A recent study demonstrated that the *S. flexneri* autotransporter *icsA* gene product can mediate bacterial cell-to-cell contact and aggregative growth in the presence of bile salts, which facilitates biofilm formation.^31^ Interestingly, the expression of *icsA* was not altered by bile salts exposure,^6^ suggesting that constitutive expression of *icsA* is sufficient to facilitate biofilm formation, possibly by interacting with other proteins or the EPS matrix.^6,29^ We hypothesize Sap either inhibits autoaggregation or that the absence of Sap in these conditions enables more efficient surface localization of other proteins such as IcsA, the adherence factors LpfA, FimA, and CsgAB, and/or EPS matrix components to aid autoaggregation during biofilm formation. In fact, it has been documented that type 1 fimbriae can block Ag43-mediated autoaggregation.^14,42^ Future peptide-specific analyses will verify these results, which should consider potential phase variation of the *sap* gene under different conditions given the homology to the Ag43 *flu* gene.^14,43^ Nevertheless, our data demonstrated that the *sap* gene product in *S. flexneri* 2457T is not directly involved in autoaggregation or biofilm formation under the conditions examined.

Despite the lack of an effect of the ∆*sap* mutation on autoaggregation and biofilm formation, the mutation affected adherence to HT-29 epithelial cells. Adherence enables pathogens to directly contact and colonize the host. Previous work by our laboratory has identified several gene products that directly facilitate *S. flexneri* adherence to colonic epithelial cells: the virulence plasmid-associated homologs *ospE1* and *ospE2*, as well as the chromosomally encoded *lpfA*, *fimA* and *csgAB* genes.^6,29,44^ The analyses here indicate that the *sap* gene product also facilitates *S. flexneri* adherence, which was not fully detected until the bacteria were subcultured with gastrointestinal signals encountered during small intestinal transit, which were provided prior to evaluating the adherence function to colonic epithelial cells. It is important to emphasize that the bile salts and glucose supplementation occur prior to adding the bacteria to the epithelial cells as previously discussed.^6,29,45^ Interestingly, the ∆*sap* mutant had the same induced adherence pattern as wild type 2457T after bile salts and glucose exposure, but the overall adherence rate was reduced relative to 2457T. Therefore, we hypothesize that the adherence function of the putative Sap protein is independent of the OspE1, OspE2, LpfA, FimA, and CsgAB proteins, which are affected by bile salts and glucose exposure.^29,44^ The results are further validated by the constitutive expression of the *sap* gene as noted in our RNA-seq analyses. Furthermore, the reduced adherence of the ∆*sap* mutant relative to 2457T provides additional evidence that the putative Sap protein is expressed on the bacterial surface, which supports the immunogenic nature of the protein^11,21^ and the increased rate of autoaggregation of the ∆*sap* mutant after EPS matrix secretion (Figure 2(b)). Future protein-specific analyses are needed to determine how the putative Sap protein is secreted and localized to the bacterial outer surface to function as an adherence factor, especially given the presence of the stop codon that potentially disrupts the association of the passenger domain with the C-terminal β-barrel domain. Finally, the ∆*sap* mutation did not affect the invasion ability of *S. flexneri*, suggesting that the virulence role of Sap is limited to epithelial cell adherence.

Sequence analysis of the *sap* gene across a collection of *S. flexneri* isolates identified a similar stop codon at nucleotide position 894 in the passenger domain of *sap* that was identified in *S. flexneri* serotype 2a strain 2457T. This stop codon is also present in the reference *S. flexneri* serotype 2a strain 301, but not present in *S. flexneri* serotype 2a strain YSH6000T. The stop codon in strain 301 resulted in a two gene annotation of the *sap* gene (SF2990) and SF2991 described as an outer membrane fluffing protein. Additionally, upon inspection of the GEMS collection of *S. flexneri* isolates, most strains either did not possess any sequence identity to the *sap* gene region or had significant sequence divergence that may result in functional variation. Of the isolates that contain the *sap* gene region, which may be specific to serotype 2a, the stop codon is in a similar location, suggesting that this annotation is a common feature of the *sap* gene in *S. flexneri*. Future analyses of the effects of this *sap* sequence divergence and genetic variability could identify effects on virulence-associated functions. Furthermore, sequence divergence could provide some protection from the host immune response.^20,26^ For Ag43, sequence and/or functional variability is well documented among the *E. coli* species, including functional diversity across different pathovars,^27^ two identical alleles in EHEC O157:H7 strain EDL933,^18^ altered functional diversity of two alleles in UPEC strain CFT073,^19^ and other variations such as Dr adhesin-mediated inhibition of Ag43 in UPEC strain IH11128.^16^ Thus, the sequence variability found in the *S. flexneri sap* gene, including the stop codon in the passenger domain of 2457T and other strains, likely affects the function of Sap across the *Shigella* species and serotypes. This new finding of virulence function related to the *sap* gene of 2457T offers opportunities to study the effects of *sap* sequence divergence on *S. flexneri* virulence.

In conclusion, this study functionally characterizes the *S. flexneri* 2457T *sap* gene. Despite the nucleotide sequence homology to the *E. coli* Ag43 *flu* gene, the pseudogene annotation in 2457T, and the lack of direct function associated with autoaggregation and biofilm formation, we demonstrated that the *sap* gene facilitates *S. flexneri* adherence to colonic epithelial cells. Furthermore, this study highlights the importance of using relevant and appropriate gastrointestinal signals to study the pathogenesis of enteropathogenic bacteria and related host–pathogen interactions. Future areas of study include protein-specific analyses and understanding of the effects of *sap* gene sequence divergence. This work improves our understanding of *S. flexneri* adherence, further demonstrates the importance of confirming proper pseudogene annotations, and establishes a new avenue of investigation to improve our understanding of *Shigella* pathogenesis and ultimately develop novel therapeutic candidates.

## Materials and methods

### Bacterial strains and growth conditions

The bacterial strains, wild type *S. flexneri* serotype 2a strain 2457T and 2457T/*sap:aph-3* (Δ*sap*, kanamycin resistance), were cultured at 37°C in LB (Lennox) or TSB. LB supplemented with glucose was added at a concentration of 2% w/v, and TSB contains approximately 0.5% w/v glucose relative to LB. Bile salts (catalog number B8756; Sigma-Aldrich) were used at a concentration of 0.4% w/v. Bacterial strains were routinely maintained on plates containing TSB with 1.5% agar and 0.025% Congo red (CR; catalog number C6277; Sigma-Aldrich). All media were autoclaved or filter-sterilized with a 0.22 µm membrane following the addition of bile salts and/or glucose. Kanamycin was used at a concentration of 50 µg/ml, as indicated.

### *Prevalence of the* sap *gene in the GEMS collection*

RNA-seq data and mapping were obtained as previously described,^6^ with read data visualized using Integrative Viewer (IGV) software (version 2.14).^46,47^ For the GEMS isolates, 806 *S. flexneri* isolates were sequenced and assembled as previously described.^24^ The *sap* gene from *S. flexneri* 2457T (GenBank Accession number NC_004741.1) was searched against the assembled genomes using BLASTN. The *sap* gene was considered significantly similar if it was > 95% nucleotide identical to > 70% of the complete *sap* gene region. Variable gene presence was defined as a BLASTN hit that was < 10^−100^ and > 95% nucleotide identity over at least 30% of the *sap* gene region. Sequences without significant similarities were considered absent.

### *Construction of the Δ*sap *mutant targeting the passenger domain of the gene*

Deletion of the 5’ signal sequence and passenger domains of the *sap* gene was performed by allelic exchange using the lambda red linear recombination method.^25^ Briefly, the plasmid pKD4 was used as a template for the amplification of the kanamycin resistance cassette (*aph-3*) via PCR using primers sap_kanF (5’-ATGAAACGACATCTGAATACCTGCTACAGGCTGGTATGGAATCACATTACGTGTAGGCTGGAGCTGCTTC-3’) and sap_kanR (5’-GTTATCGGGAATATTCCAGGTGGCACCAGAGGCGAGAGTG ACATTCGTGGCATATGAATACCTCCTTAG-3’). Each primer consisted of a 50-nucleotide sequence homologous to the specified region of *sap*, followed by 20 nucleotides homologous to *aph-3* at the 3’ end. The PCR product was then used to transform *S. flexneri* 2457T harboring plasmid pKM208, which contains the λ-red *gam, beta*, and *exo* genes, to facilitate the homologous recombination of the linear PCR product into the *sap* region. Positive transformant colonies were selected by kanamycin resistance and confirmed via PCR using primers sap_conF (5’-CCTGCCGGTATCCACATCTG-3’) and sap_conR (5’-TGCCATATCCGGGCGTACAC-3’), which amplified the 5’ upstream region of *sap* and into the kanamycin cassette to generate a 1.3 kb product to confirm presence of the mutation. Wild type 2457T was used as the negative control for the PCR. Finally, to confirm the ∆*sap* mutation was the only significant sequence alteration in the ∆*sap* mutant strain, total genomic DNA was extracted and sequenced at SeqCenter, Pittsburgh, PA. The *sap* mutant was evaluated using BreSeq^48^ with default settings with Illumina generated 2 × 151bp paired-end read data as the input for variant calling against the *S. flexneri* 2457T reference genome (CP100044-CP100048).

### Analysis of bacterial cell aggregation

To analyze the possible role of the *sap* gene product in bacterial cell aggregation, or autoaggregation, the clumping assay was performed as previously described with minor modifications.^32^ Briefly, overnight cultures of wild type 2457T and the Δ*sap* mutant were standardized and subcultured 1:50 into either LB with 2% w/v glucose or TSB media, each with or without 0.4% w/v bile salts. Cultures were grown at 37°C at 225 rpm for approximately 2.0 to 2.5 h to an OD_600_ of 0.7. For analysis of autoaggregation after biofilm formation, cultures were grown for 4 h, and the OD_600_ values were measured. Bacterial subcultures were then centrifuged at 4000 × *g*, washed, resuspended in 2 ml of 1X PBS, and standardized to an OD_600_ of 1.0 in polyethylene tubes. The tubes were then placed on a benchtop at room temperature for up to 18 h. The OD_600_ of the culture supernatants was measured by taking 100 µl at a distance of 0.5 cm below the surface of the culture. Measurements were taken at the initial placement of the culture tubes on the benchtop (time 0) and at each time point. The supernatants were placed in a 96-well plate for absorbance readings using a SpectraMax® plate reader. The final cellular aggregation was measured by subtracting the OD_600_ at time 0 from the OD_600_ at the end of the assay.

### Analysis of biofilm formation

To evaluate the role of the *sap* gene product in biofilm formation, a biofilm assay was performed in both LB supplemented with glucose and TSB, both with and without bile salts, as previously described.^6,30^ Briefly, overnight cultures of wild type 2457T and the Δ*sap* mutant were standardized and 20 µL of each culture was added to 1 ml of each media formulation (1:50 dilution). Next, 130 µL of the inoculated culture was added to three wells (for technical triplicate readings) of a flat-bottomed, tissue culture-treated 96-well plate (Millipore Sigma, USA). Plates were incubated at 37°C, and biofilm formation was measured every 30 min between 2 and 4 h of incubation. Afterward, the media were carefully aspirated, and the plates were washed with 1X PBS and left to dry. The wells were then stained with 0.5% crystal violet for 5 min, washed five times with distilled water, and incubated with 95% ethanol for 30 min at room temperature. The absorbance was measured at an optical density of 590 nm (OD_590_) using a SpectraMax® plate reader. Samples were blanked to wells that were incubated with media and treated using the same procedure.

### HT-29 adherence and invasion assays

The role of the *sap* gene product in the adherence and invasion of *S. flexneri* was evaluated using the human colorectal adenocarcinoma cell line HT-29 (ATCC HTB-38) as previously described.^6^ Briefly, HT-29 cells were cultured in 6-well plates using DMEM with 10% heat-inactivated fetal bovine serum (Thermo Fisher Scientific, Waltham, MA, USA) at 37°C under 5% CO_2_ until reaching approximately 85% confluency. For the adherence assay, overnight cultures of wild type 2457T and the Δ*sap* mutant were diluted 1:50 and subcultured in LB supplemented with 2% w/v glucose or TSB media, both with or without 0.4% w/v bile salts. Subcultures were grown at 37°C with shaking at 225 rpm for approximately 2 h until the cultures reached an OD_600_ of 0.7. The subcultures were then standardized to an OD_600_ of 0.35, centrifuged, washed with 1X PBS, and resuspended in 1 ml of DMEM for application onto HT-29 cells. Prior to the application of the bacterial cultures, HT-29 cells were washed in 1X PBS and resuspended in DMEM without FBS. For application of the bacteria onto the cells, the DMEM was removed, and the standardized bacterial samples resuspended in 1 ml of DMEM were added to the cells. The cells were incubated at 37°C with 5% CO_2_ for 3 h. Afterward, monolayers were washed five times with 1X PBS and the cells were subsequently lysed with 1% Triton X-100. The number of adherent bacteria was determined by serial dilution and colony counting on TSB agar plates containing CR. The adherence rate was calculated as [recovered bacterial titer/infecting bacterial titer] × 100%.

For the invasion assay, bacteria were prepared as described above. After applying the bacteria onto HT-29 cells, an additional 1 ml of DMEM was added to each well, and the plates were centrifuged at 3000 rpm for 10 min to facilitate bacterial contact with the cells. Afterward, the plates were incubated for 45 min at 37°C with 5% CO_2_. The HT-29 cells were then washed five times with 1X PBS and incubated with DMEM plus 50 µg/ml gentamicin at 37°C with 5% CO_2_ for 30 min. The cells were washed again with 1X PBS and incubated with fresh DMEM plus gentamicin for 60 min to ensure lysis of extracellular bacteria. After the incubation, HT-29 cells were washed three times with 1X PBS, and the cells were lysed with 1% Triton X-100. The number of invading bacteria was determined by serial dilution and colony counting on TSB agar plates containing CR. The invasion rate was calculated as [recovery bacterial titer/infecting bacterial titer] × 100%.

### Statistical analyses

All experiments were performed with three biological replicates, with either three technical replicates for the biofilm formation assay or two technical replicates for the autoaggregation and infection (adherence and invasion) assays. Data for the infection assays were plotted relative to 2457T from the LB + 2% glucose + 0.4% bile salts or TSB + 0.4% bile salts subcultures, which were set at 100%. Two-way analysis of variance (ANOVA) was used to evaluate differences between treatments and/or wild type 2457T and Δ*sap* mutant in the various assays. Graphs and statistical comparisons were generated using GraphPad Prism 5.04. Statistical significance was set at *p* value of < 0.05.

## Data Availability

The authors confirm that data supporting the findings of this study are available within the article or upon request from the corresponding author.
